# Patient resuscitated after cardiopulmonary arrest exhibits abnormally increased phenytoin metabolic rate due to unknown factors: a case report

**DOI:** 10.1186/s40780-024-00374-6

**Published:** 2024-08-28

**Authors:** Ayumu Nagamine, Takuya Araki, Hideaki Yashima, Kiyohiro Oshima, Kyoko Obayashi, Koujirou Yamamoto

**Affiliations:** 1 Education Center for Clinical Pharmacy, Faculty of Pharmacy, Takasaki University of Health and Welfare, 60 Nakaorui-Machi, Takasaki, Gunma 370-0033 Japan; 2 Department of Clinical Pharmacology and Therapeutics, Gunma University Graduate School of Medicine, 3-39-22 Showa-Machi, Maebashi, Gunma 371-8511 Japan; 3 Department of Emergency Medicine, Gunma University Graduate School of Medicine, 3-39-22 Showa-Machi, Maebashi, Gunma 371-8511 Japan

**Keywords:** Fosphenytoin, Phenytoin, Therapeutic drug monitoring, Pharmacokinetics, Cardiopulmonary arrest

## Abstract

**Background:**

Fosphenytoin (FOS) is a prodrug of phenytoin (PHT) with a metabolism that exhibits Michaelis–Menten-type kinetics. Genetic polymorphisms of the metabolic enzymes of PHT make it challenging to predict its plasma concentrations. High plasma PHT concentrations are typically problematic, and several causes have been elucidated. In contrast, cases of patients with low PHT plasma concentrations that did not increase despite the administration of appropriate PHT doses have been reported, and the causes may include changes in plasma protein-binding rates, genetic mutations, and concomitant use of drugs that induce liver enzymes; however, even these factors do not explain the low PHT plasma concentrations in some cases.

**Case presentation:**

We encountered the case of a patient with plasma PHT concentrations that were continuously < 0.7 µg/mL after daily use of FOS for seizures that occurred after cardiopulmonary arrest. We analyzed the protein-unbound fraction, urinary metabolites, and related genes to investigate the cause. False negatives due to the measurement method, errors in dosage and administration method, and increased excretion of PHT were excluded. Hepatic metabolic activity of PHT increased to 4.6–6.1 times the normal level. The S/R ratio of 5-(p-hydroxyphenyl)-5-phenylhydantoin-glucuronide, a major PHT metabolite, was normal at 15.2, suggesting increased activities of CYP2C9 and CYP2C19. Furthermore, the protein-unbound fraction of PHT was 5.2–6.9%, CYP2C19^*^17 was wild type, and there was no concomitant drug use to induce both enzymes.

**Conclusions:**

The low PHT plasma concentration in this patient was found to be caused by increased hepatic metabolic activity that could not be explained by known factors. Careful monitoring is necessary to consider the possibility of increased hepatic metabolic activity in similar cases.

## Background

Fosphenytoin (FOS) is a water-soluble prodrug of phenytoin (PHT) that is widely used to treat conditions such as status epilepticus [1, 2]. PHT is metabolized to 5-(p-hydroxyphenyl)-5-phenylhydantoin (p-HPPH) by CYP2C9 and CYP2C19. Because of genetic mutations in these metabolizing enzymes, there are large individual differences in plasma levels of PHT. Furthermore, since PHT metabolism exhibits Michaelis–Menten pharmacokinetics [3], it is difficult to control plasma PHT concentrations. Therefore, a detailed dosage protocol for FOS based on therapeutic drug monitoring (TDM) is recommended, and the total PHT plasma concentration should be adjusted to 10–20 µg/mL (1–2 µg/mL as free PHT) to promote the drug’s efficacy and limit its side effects [3, 4]. During TDM of PHT, attention is often focused on the rapid increase in plasma PHT concentrations due to saturation of drug metabolism. However, some patients exhibit extremely low PHT trough concentrations (< 5 µg/mL) even after the administration of clinically recommended doses of PHT of 5–10 mg/kg/day [5, 6]. Factors contributing to low PHT plasma concentrations may include decreased protein binding [7–11], *CYP2C19* rs12248560 mutation [12], and concomitant use of liver enzyme inducers for metabolic enzymes [13, 14]; nevertheless, even these factors cannot explain the low PHT plasma concentration in some cases. In the present study, we encountered the case of a patient with plasma PHT concentrations that were continuously below the limit of quantification after daily use of FOS for seizures that occurred after cardiopulmonary arrest. We analyzed the patient’s plasma PHT concentration, protein-unbound fraction of plasma PHT, urinary PHT metabolite concentrations, and related genes to investigate the factors responsible for the abnormally low plasma PHT concentrations.

## Case presentation

A 55-year-old man (height: 175 cm; weight: 76.4 kg) was found in cardiopulmonary arrest by hanging and was transported by ambulance (day 0) to our institution. On admission, although resuscitation was performed, he remained in a coma due to hypoxic encephalopathy, and mechanical ventilation and 24-h therapeutic hypothermia were initiated. His vital signs were as follows: temperature, 35.1 °C; heart rate, 146 bpm; blood pressure, 170/90 mmHg; and Glasgow Coma Scale score, 3. The patient also exhibited generalized convulsions. No abnormal laboratory values, such as renal function indicator levels or electrolyte levels, were identified on admission, except for slightly elevated levels of aspartate aminotransferase (AST) at 86 U/L, alanine aminotransferase (ALT) at 60 U/L, alkaline phosphatase (ALP) at 341 U/L, and gamma-glutamyl transpeptidase (γ-GTP) at 122 U/L. Plain computed tomography revealed cerebral edema. Interviews with family members revealed no anamnesis, history of allergies, or medication use.

**Fig. 1:**
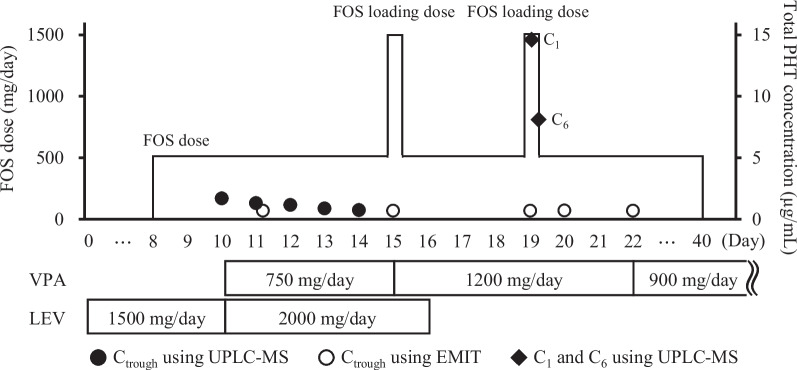
Change in plasma PHT concentrations and the dose adjustments of anticonvulsants. Day 0 was the day of admission, and 500 mg of FOS was administered intravenously once daily from days 8 to 40, with an additional 1000 mg loading dose (total 1500 mg) administered on days 15 and 19. Closed circles indicate plasma PHT concentrations (C_trough_) obtained using UPLC-MS, and open circles indicate plasma PHT concentrations (C_trough_) obtained using EMIT. Closed diamonds are plasma concentrations of PHT at 1 h (C_1_) and 6 h (C_6_) after the loading dose, which were analyzed using UPLC-MS. EMIT, enzyme-multiplied immunoassay technique; FOS, fosphenytoin; LEV, levetiracetam; PHT, phenytoin; UPLC-MS, ultra-performance liquid chromatography-mass spectrometry; VPA, sodium valproate

After admission, the patient received continuous intravenous infusions of fentanyl (days 0 − 13), midazolam (days 0 − 12), propofol (days 3 − 12), and sodium thiopental (days 5 − 9) for sedation. In addition, 750 mg of levetiracetam (LEV) twice per day (intravenous [IV] infusion over 15 min) was initiated as an anticonvulsant on day 0. Due to poor seizure control, 500 mg of FOS once per day (IV over 30 min) was added starting on day 8; LEV was increased to 1000 mg twice per day (IV over 15 min) starting on day 10; and 250 mg of sodium valproate (VPA) by nasogastric (NG) tube, three times per day, was added on the same day. Figure 1 illustrates the dose adjustments of the anticonvulsants, FOS, VPA, and LEV, as well as the changes in plasma PHT concentrations. Thereafter, the frequency of seizures decreased, and the sedative medications were discontinued. However, because the plasma PHT trough concentration on day 15 was below the limit of quantification (0.7 μg/mL using the enzyme-multiplied immunoassay technique [EMIT]), 1500 mg of FOS was administered as the loading dose. Furthermore, given that the plasma VPA trough concentration was 25 µg/mL, which was below the target concentration range (50–100 µg/mL), the dose of VPA was increased to 400 mg by NG tube, three times per day, the same day. Additionally, as elevated liver function indicator levels (AST: 194 IU/L, ALT: 245 IU/L, ALP: 1390 U/L, γ-GTP: 649 U/L) were confirmed on day 15, LEV was discontinued from day 16. Subsequently, the patient’s liver function was improved, and no worsening of seizures was observed. Because the plasma PHT trough concentration on day 19 was below the limit of quantitation, a loading dose of 1500 mg of FOS was administered; however, the abnormally low plasma PHT trough concentration persisted. Although the VPA trough concentration on day 19 (39 µg/mL) was lower than the target concentration range, the dose was continued because the seizures had subsided. The dose was reduced to 300 mg by NG tube, three times per day, on day 22. Subsequently, FOS was discontinued on day 40 because there were no seizures; the patient remained unconscious and was transferred to another hospital.

Plasma PHT concentrations were analyzed with ultra-performance liquid chromatography-mass spectrometry (UPLC-MS) using plasma residue collected during hospitalization. On days 10 − 14, plasma PHT concentrations decreased and were similar to those measured using the EMIT method (Fig. 1). Protein-free PHT concentrations were analyzed using ultrafiltration, and the protein-unbound fraction on days 10 − 14 was 5.2–6.9%. Serum albumin levels were 2.3, 2.2, 1.9, 2.1, and 2.3 mg/dL on days 8, 10, 11, 12, and 14, respectively, which increased to over 3.0 mg/dL after day 26. PHT concentrations at 1 and 6 h after the initiation of the loading dose (C_1_ and C_6_, respectively) on day 19 were 14.6 and 8.1 μg/mL, respectively. Assuming a half-life of 15 min for FOS, based on a previous study involving healthy Japanese men aged 20 − 40 years [15], the elimination rate constant of FOS was calculated to be 0.0462 min^−1^, and it was estimated, using Eqs. 1 and 2, that approximately 86.5% of the FOS was converted to PHT 1 h after the start of administration. Moreover, since 1500 mg of FOS is equivalent to 931.5 mg of PHT, the PHT at C_1_ was converted to 805.7 mg, and the volume of distribution (V_d_) of PHT from C_1_ was estimated to be 55.2 L. Using Eq. 3, the PHT clearance of t_1–6_ and t_6–24_ calculated from C_1_ and C_6_ on day 19 and the PHT concentration at 24 h after commencing treatment (C_24_: 0.70 μg/mL) were 6.50 and 7.50 L/h, respectively. The equations employed for the above calculations are as follows:

1 $$X=\frac{{k}_{0}}{{k}_{e}}\bullet \left(1-{exp}^{-{k}_{e}\bullet t}\right) \left(t\le {t}_{0}\right)$$

2 $$X={X}_{0}\bullet {exp}^{-{k}_{e}\bullet \left(t-{t}_{0}\right)} \left(t>{t}_{0}\right)$$

3 $$C={C}_{0}\bullet {exp}^{-{k}_{e}\bullet t}$$

where *X* is the amount of FOS in the body; *X*_*0*_ is the amount of FOS in the body at the end of infusion; *k*_*0*_ is the infusion rate (50 mg/min); *k*_*e*_ is the elimination rate constant; *t* is the time after the start of administration; *t*_*0*_ is the duration of infusion (30 min); *C* is the PHT concentration of t_6_ or t_24_; and *C*_*0*_ is the PHT concentration of t_1_ or t_6_.

Urine samples were collected for 24 h immediately before FOS administration on day 30 to before FOS administration on day 31. Urinary metabolites were evaluated using UPLC-MS, which yielded the recovery of metabolites corresponding to almost the entire quantity of FOS administered (101.8%). Of the FOS-derived constituents recovered from the urine samples, 96.8% were p-HPPH-glucuronide with an S/R ratio of 15.2, and 5% were PHT-glucuronide. Additionally, genetic testing revealed that CYP2C19^*^17 was wild type.

## Discussion and Conclusions

In this case, 500 mg of FOS was administered daily to the patient to control his generalized convulsions; however, the patient’s plasma PHT trough concentration was abnormally low and was below the limit of quantification (0.7 µg/mL) in the EMIT assay. Factors contributing to low plasma trough concentrations of PHT may include false negatives in the EMIT assay, incorrect FOS dosage or administration, or increased PHT clearance. The EMIT method, which is widely used in clinical practice, can produce false negatives due to interference by endogenous proteins, such as immunoglobulins. However, in the present case, a false negative in the EMIT assay was excluded by remeasurement using UPLC-MS. Urinary excretion was evaluated to confirm the dosage of FOS that was administered to the patient. p-HPPH-glucuronide, equivalent to 96.8% of the theoretical dose of FOS, was detected in the urine, consistent with findings from previous reports [16–18]. This confirmed that FOS was accurately administered to the patient according to the indicated dosage, thereby excluding the possibility of low plasma PHT concentrations due to dosage or administration errors. Next, we investigated the potential for increased PHT clearance. PHT clearance was calculated in accordance with the Michaelis–Menten equation (Eq. 4), as follows:

4 $$\frac{{dC}_{t}}{{d}_{t}}=\frac{{V}_{max}\bullet {C}_{t}}{{K}_{m}+{C}_{t}}$$

where *V*_*max*_ represents the decay of the plasma PHT concentration when elimination is saturated, and *K*_*m*_ is the plasma PHT concentration at 50% saturation. Using K_m_ (4.26 μg/mL) and V_max_ (6.31 mg/kg/day), which are the parameters of PHT metabolism in adult Japanese patients with epilepsy who have good seizure control [19], the PHT clearances at plasma PHT concentrations of 14.6 and 8.1 µg/mL were 1.07 and 1.63 L/h, respectively. This indicated that the estimated PHT clearances of 6.50 and 7.50 L/h in this patient were approximately 4.6–6.1 times higher than the typical PHT clearances.

The increased clearance of PHT may be due to elevated excretion of PHT itself or to an increased rate of PHT liver metabolism. In the present case, increased excretion of PHT itself was ruled out because 96.8% of the administered dose was detected as p-HPPH-glucuronide and only 5% as PHT-glucuronide in the urine. PHT is metabolized by hepatic intrinsic clearance, and its metabolic activity is greatly influenced by changes in the protein-unbound fraction [7–11], genetic polymorphisms [12], and enzyme induction and inhibition by concomitant drugs [13, 14]. Among the patient’s concomitant medications, fentanyl, midazolam, propofol, sodium thiopental, and LEV did not cause CYP induction or alter protein binding to PHT, and no interactions related to increased PHT metabolism were identified. In contrast, VPA decreases the total PHT concentration, primarily by increasing the protein-unbound fraction of PHT. Furthermore, the patient had hypoalbuminemia, suggesting that the increased unbound fraction contributed to the increased PHT metabolism. However, the unbound fraction of PHT in our patient was 5.2–6.9%, which did not increase from the unbound fraction of approximately 10% for typical PHT [3]. Therefore, the contribution of the concomitant use of VPA to the hypermetabolism of PHT in the present case was considered insignificant, and drug interactions were not considered to be the cause of the low plasma PHT concentrations in this patient. Moreover, although CYP2C19^*^17 is a factor that increases the metabolic activity of PHT [12], this patient did not possess the ^*^17 allele. Because p-HPPH, a metabolite of PHT, is produced by CYP2C9 as the S-isomer and by CYP2C19 as the R-isomer, the contribution of the metabolizing enzyme can be evaluated from the S/R ratio [20]. The S/R ratio of p-HPPH-glucuronide in this patient was 15.2, which is consistent with that reported by Argikar et al. [20], confirming that the contribution of CYP2C9 and CYP2C19 to PHT metabolism was normal. Hence, the increased hepatic metabolic activity of PHT in this patient could not be explained by any known factor and was believed to be due to an equal increase in the activity of at least two enzymes, CYP2C9 and CYP2C19. Furthermore, considering that the plasma VPA concentration, which undergoes hepatic metabolism by glucuronidation, was also low, it cannot be excluded that the increased activity of various enzymes involved in glucuronidation may have played a role.

We considered three hypotheses that may explain the abnormal increase in the PHT hepatic metabolic activity in this patient. First, the patient may have a certain condition, such as post-cardiac arrest syndrome, after resuscitation from cardiopulmonary arrest. Post-cardiac arrest syndrome is a “sepsis-like” syndrome in which plasma levels of various inflammatory cytokines and endotoxins increase following cardiopulmonary resuscitation [21]. As increased plasma levels of inflammatory cytokines decrease CYP expression and activity [22], elevated concentrations are usually problematic. Furthermore, therapeutic hypothermia, which is often administered after cardiac arrest, has also been noted as a potential source of decreased drug clearance due to decreased CYP expression [23]. However, the pathogenesis after cardiopulmonary arrest remains unclear, including possible changes in drug metabolism capacity. As cytokines could not be analyzed in the present case due to the patient’s plasma volume, a detailed analysis in a similar case is required. Second, the effect of daily administration of FOS was considered. Ohno et al. [24] reported that daily treatment with FOS for ≥ 2 days resulted in a gradual decrease in plasma PHT concentrations and an increased risk of seizures in patients with autoimmune-mediated encephalopathy accompanied by reflux disease and/or ileus. Although the cause of the decrease in plasma PHT concentration due to the daily administration of FOS has not been identified, we observed a gradual decrease in the PHT concentration after day 10, which was the third day of FOS administration, suggesting a possible effect of daily FOS administration. Third, the expression levels and metabolic activities of several metabolic enzymes, such as CYP2C9 and CYP2C19, may have increased due to unknown genetic polymorphisms or other factors.

This study had several limitations. First, because the remaining blood from the laboratory tests was used, an optimal blood collection point for pharmacokinetic prediction could not be obtained, and the half-life of FOS, as well as K_m_ and V_max_ for PHT in this patient, could not be calculated. Therefore, the half-life of FOS was obtained from healthy Japanese adult males included in the study by Inoue et al. [15], and K_m_ and V_max_ for PHT were derived from a population of Japanese epilepsy patients with well-controlled seizures in the study by Yukawa et al. [19]. Inoue et al. [15] sought clearance of FOS with a single dose of 375–750 mg of FOS, with a dosing time of 30 min. Yukawa et al. [19] included epilepsy patients without CYP2C19^*^2 and *3 alleles, which are associated with reduced PHT metabolic activity, and also included patients taking concomitant medications, such as carbamazepine, phenobarbital, and VPA. Our patient experienced uncontrolled seizures due to hypoxemia, and the pharmacokinetics of PHT may differ from those in healthy adult males and patients with epilepsy. Furthermore, the concomitant use of CYP inducers, such as carbamazepine, may increase the V_max_ of PHT. As patients with uncontrolled seizures, like ours, are often encountered in the emergency department, establishing pharmacokinetic parameters in this patient population may contribute to understanding the pathogenesis of hepatic metabolic hyperactivity. Second, as it has not been confirmed whether all FOS formulations were in the same lot, variations among the formulations cannot be excluded.

In conclusion, by measuring plasma and urine PHT and metabolite concentrations in a patient with abnormally low plasma PHT concentrations, we report, for the first time, a case of increased hepatic metabolic activity that could not be explained by known factors. This increase in hepatic metabolic activity may be due to the increased expression or activity of several enzymes, including CYP2C9 and CYP2C19. In similar cases, such as after cardiopulmonary arrest, more detailed drug management that takes into consideration the increase in hepatic metabolic activity is important. Specifically, if increased hepatic metabolic activity is detected by assessing not only the PHT trough concentration but also the PHT peak concentration, an individualized dosing design is desirable, such as administering PHT multiple times a day after confirming that there is no accumulation of PHT metabolites. In addition, because such patients may also have increased metabolism of other hepato-metabolized drugs, individual dosing regimens based on TDM should also be considered for concomitant medications. A detailed analysis of similar cases to elucidate the cause of the increased hepatic metabolic activity will improve the understanding of PHT metabolism and contribute to safer drug therapy.

## Data Availability

The datasets generated and/or analyzed during the current study are available from the corresponding author on reasonable request.

## Abbreviations

ALP: Alkaline phosphatase
ALT: Alanine aminotransferase
AST: Aspartate aminotransferase
EMIT: Enzyme-multiplied immunoassay technique
FOS: Fosphenytoin
IV: Intravenous
LEV: Levetiracetam
NG: Nasogastric
p-HPPH: 5-(P-hydroxyphenyl)-5-phenylhydantoin
PHT: Phenytoin
TDM: Therapeutic drug monitoring
UPLC-MS: Ultra-performance liquid chromatography-mass spectrometry
V_d_: Volume of distribution
VPA: Sodium valproate
γ-GTP: Gamma-glutamyl transpeptidase
